# Hormone synergy: Auxin and jasmonate boost abscisic acid signaling via ARF10 and ARF16

**DOI:** 10.1093/plcell/koad012

**Published:** 2023-01-18

**Authors:** Carlisle Bascom

**Affiliations:** Assistant Features Editor, The Plant Cell, American Society of Plant Biologists, USA; Department of Cell and Developmental Biology, University of California San Diego, La Jolla, California 92093, USA

**Figure 1:**
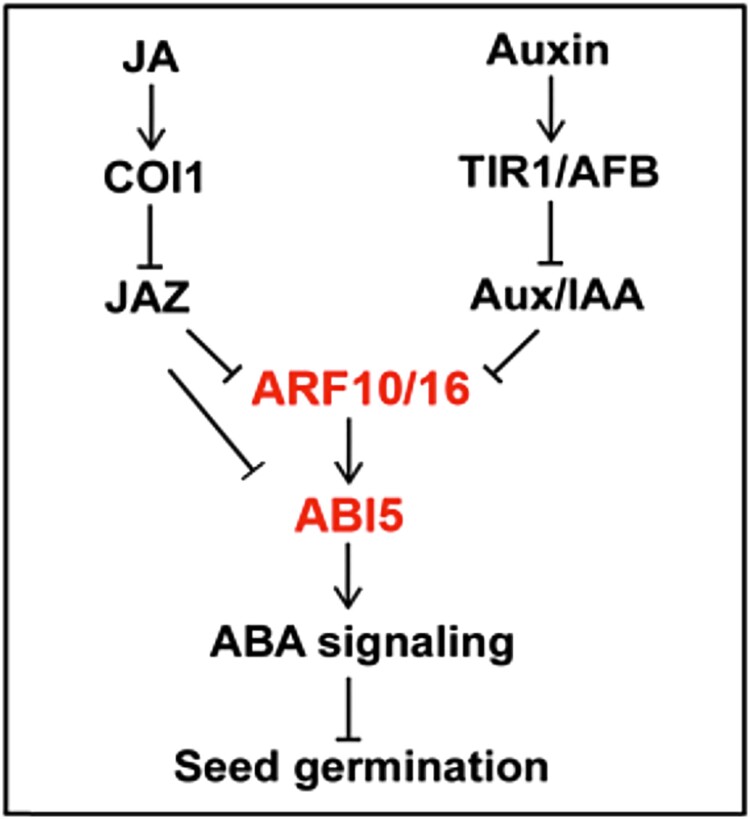
Model for the synergistic effects of JA and IAA on ABA signaling. JAZs repress both ARF10/16 and ABI5 transcription factors. ARF10/16 is repressed in the canonical auxin signaling pathway. As levels of auxin and JA rise with ABA, all three pathways work to supercharge ABA signaling. Adapted from Mei et al. (2022).

Plants integrate stimuli via hormones to respond to developmental cues as well as environmental conditions. Previous investigations showed that the signaling pathways of the phytohormones jasmonate and auxin can each affect the output of the phytohormone abscisic acid (ABA). ABA attenuates several aspects of plant growth, including germination. In the presence of ABA, transcription factors such as ABSCISIC ACID INSENSITIVE5 (ABI5) up-regulate ABA-responsive genes. Previous work found that AUXIN RESPONSE FACTORS (ARFs) 10 and 16, transcription factors that are derepressed upon auxin signaling, function as positive regulators of ABA signaling (Liu et al., 2013). In addition to auxin, jasmonate (JA) enhances ABA signaling. JASMONATE ZIM-DOMAIN (JAZ) proteins repress activators of jasmonate-sensitive genes and are degraded as jasmonate levels rise. And interestingly, *jaz* mutants are hypersensitive to ABA (Pan et al., 2020). Indeed, exogenous methyl jasmonate (MeJA) or auxin (IAA) application enhances the effects of ABA cotreatment. While auxin and jasmonate affect the ABA signaling pathway individually, whether IAA and MeJA have synergistic effects with ABA was largely unknown and there was no clear unified molecular mechanism for IAA–JA–ABA cross-talk. In this issue of *The Plant Cell,* Song Mei and coauthors (Mei et al., 2022) use pharmacological, genetic, and biochemical techniques to elucidate a nexus of auxin, jasmonate, and ABA signaling pathways acting together to control seed germination (see Figure 1).

To illustrate that auxin and jasmonate work synergistically to enhance ABA signaling, the authors measured *Arabidopsis* germination on media with different combinations of the three phytohormones. Alone and together, 1 µM IAA and 10 µM MeJA had little effect on germination rates. Yet each enhanced the ability of 0.3 µM ABA to inhibit germination. Consistent with an additive effect, the application of all three hormones reduced the germination rate more than IAA or MeJA paired with ABA alone. Additionally, mutants with less endogenous IAA were resistant to the effects of combined MeJA–ABA treatment. Meanwhile, IAA overproducing plants grown on medium with both MeJA and ABA phenocopied wild-type plants treated with all three hormones. This synergy requires the complete auxin signaling pathway. Indeed, mutants deficient in auxin signaling are resistant to MeJA–ABA cotreatment.

Having established the strength of hormone synergy, the authors sought to identify the proteins that connect these three signaling pathways, using JAZ1 in a yeast-two-hybrid screen. Interestingly, they recovered ARF16. Indeed, both ARF16, and the close homolog ARF10, interact with several members of the JAZ family (see Figure 1). Recent work demonstrated that JAZ proteins also interact with ABI5, an ABA-sensitive transcription factor (Ju et al., 2019; Pan et al., 2020). Subsequent analyses revealed that both ARF10 and ARF16 interact with JAZ1, JAZ4, JAZ7, JAZ9, and ABI5. This remarkable discovery corroborates the pharmacological data suggesting that both MeJA-induced degradation of JAZ proteins and IAA-induced derepression of the ARFs enhance the transcriptional activity of ABI5 (see Figure 1).

The authors then sought to validate the hypothesis that ARF-ABI5 interaction directly up-regulates ABA-sensitive genes and that this activity is repressed by JAZ proteins. The authors employed dual-luciferase assays with an ABA-responsive promoter to investigate the effect of each protein on ABA-induced expression. Corroborating the genetic data, both ARFs enhanced the transcriptional activity of ABI5. Additionally, JAZ1 repressed ABI5 activity. All three together produce a moderate transcriptional response, attenuated from ABI5 alone. Taken together, the data presented by Mei et al. establish a concise molecular model by which jasmonate, auxin, and ABA are integrated into seed germination via JAZ and ARF16/10 interacting with ABI5.
